# Continental philosophical perspectives on life sciences and emerging technologies

**DOI:** 10.1186/s40504-016-0041-7

**Published:** 2016-06-13

**Authors:** Hub Zwart, Laurens Landeweerd, Pieter Lemmens

**Affiliations:** Department of Philosophy and Science Studies, Faculty of Science, Institute for Science, Innovation and Society (ISIS), Radboud University Nijmegen (the Netherlands), p.p. box 9010, 6500 GL Nijmegen, the Netherlands

## Aims and scope of the thematic series

Life sciences and emerging technologies raise a plethora of issues. Besides practical, bioethical and policy issues, they have broader, cultural implications as well, affecting and reflecting our zeitgeist and world-view, challenging our understanding of life, nature and ourselves as human beings, and reframing the human condition on a planetary scale. In accordance with the aims and scope of the journal, LSSP aims to foster engaged scholarship into the societal dimensions of emerging life sciences (Chadwick and Zwart 2013) and via this thematic series, the journal provides a podium for authors who intend to address concrete issues from a ‘continental philosophical’ perspective, which may include (post)phenomenology, hermeneutics, dialectics, (post)structuralism, psychoanalysis, critical theory and similar approaches. The series aims to contribute to a diagnostics of the present and a prognostics of the future, focusing on critical normative challenges (such as embodiment, intimate technologies, social justice, biopower, nanomedicine, human enhancement and the anthropocene) and building on the work of key authors such as Hegel (1830), Heidegger (1953, 1953/1954), Bachelard (1938), Canguilhem (1975), Lacan (1966, 1969-1970/1991), Habermas (1968), Serres (1972), Foucault (1969), Žižek (2006/2009), Stiegler (2010), Sloterdijk (2001, 2009) and others, but targeting concrete up-to-date events and case studies against the backdrop of broader developments within the techno-scientific culture. Rather than as a euro-centric position, we aim to develop continental perspectives in interaction with moral deliberations currently unfolding on a truly global scale. Finally, special attention is given to genres of the imagination (novels, movies, theatre, art) as laboratories for reflection (Zwart 2014, 2016b).

## ‘Continental’ perspectives

Although the signifier ‘continental philosophy’ began its career as a pejorative term and remains difficult to define exactly, a common profile is nonetheless discernible among adherents (cf. Critchley 2001; Glendinning 2006). The authors mentioned above share a certain style of thinking, a common set of intellectual challenges and ideas. A family likeness can be discerned among their oeuvres. Although the authors themselves (and the scholars studying their work) often highlight the singularities of these oeuvres and the differences with other (previous or contemporary) thinkers, this emphasis on dissension may obfuscate the common ground: the discursive landscape or ambiance in which they all dwell, as voices in an unfolding dynamics of thought, engaged in a “lively, dialectical relationship with the world” (Anderson et al. 1968). Continental philosophers share the conviction, moreover, that science is not the only reliable or meaningful access to reality and they generally embrace epistemological pluralism. There are other revealing ways of experiencing and disclosing the world, such as various societal practices or art. Moreover, science as a form of world-disclosure is profoundly historical, expressing and reflecting the zeitgeist of an epoch, co-evolving with technological developments and contributing to a particular style of thinking. Also, continental philosophy grants an active role to human existence and praxis in shaping the world. Especially science is seen as a transformative practice: not only exploring, but also interacting with and reorganising the world. And continental philosophers implicitly or explicitly endorse the claim that the basic objective of philosophy is to develop a diagnostics of the present, against the backdrop of a broad temporal horizon, so that a diagnostics inevitably also involves an anamnesis of the past and a prognostic of the future. Finally, they tend to agree that we currently witness an epoch of profound disruption, of political and scientific revolutions, spreading into other realms of culture, giving rise to a metaphysical mutation. It is the objective of philosophers not only to probe and assess the profile of this transition (to capture the newly emerging zeitgeist in thought, as Hegel phrased it), but also to act as midwifes in the Socratic sense.

## Themes and topics

There are a number of themes and topics which we would like to highlight explicitly as arenas for reflection:

1. Digitalisation. By this we mean the impact of digital tools and instruments on the pace and scale of life sciences research and on the ways in which life scientific research is conducted in terms of ambitions, methods and transdisciplinary collaborations. We would like to explore and assess the extent to which digitalisation has resulted in the emergence a new paradigm (Kuhn) or episteme (Foucault) in the terabyte age of big data research (Zwart 2016a). Notably, we want to highlight the impact of digitalisation on the position of the researchers (marginalisation and anonymisation on the one hand, compensated by the cultivation of “responsible research” on the other). Also, we want to explore and foster opportunities provided by new digital platforms for re-initiating the dialogue between life sciences and the humanities (bridging the ‘two worlds’ divide).
2. Synthetic biology: taking us from reading to rewriting genomes (Zwart 2012) and from describing the biological real to the operational practices of biotechnology (Hottois 1990), resulted in new transdisciplinary areas for research, from biomaterials and biomimicry up to the synthetic cell. These events not only open up a plethora of (beneficial or risky) innovations (Osseweijer et al. 2010), but also raise issues and questions on the ontological level, such as: the synthetic cell as a metaphysical turn or mutation, opening up a new chapter in the history of life and challenging established understandings of life, nature, technology and the role and place of human beings. On the one hand, the synthetic turn triggers awareness of the complexity of living systems, but at the same time life itself seems to become modifiable even on the molecular level. How to assess the philosophical profile of this scientific revolution?
3. Cognitive enhancement. Although emerging technologies in the neurosciences are often initially focussed on disabled individuals, this type of research basically aims at deepening our understanding of cognitive and sensory processes. Bio-electronic gadgets may allow paraplegic patients to walk again, but such devices may also ‘optimise’ what we now regard as normal functioning (Hildevoorde and Landeweerd 2010). Non-invasive stimulation devices such as tDCS, TMS, tFUS may help individuals to achieve every-day goals such as mobility, multi-sensory integration, computer-use, paying attention, relaxation, falling asleep, improving one’s gaming skills or learning. They may increasingly be used in the every-day lifeworld to optimise brain processes in ‘normal’ people who are not impaired by mental illness or cognitive disorders, boosting the ‘plasticity’ of sensory and neural systems. A continental approach moves away from neuro-centric approaches to address how such gadgets may affect our sense of embodiment and being-in-the-world, against the backdrop of broader socio-cultural developments such as cognitive capitalism and emerging collaborations and/or increasing tensions between ICT devices and humans (Lemmens 2015a, Lemmens 2015b).

We hereby cordially invite (teams of) authors who want to contribute to the aims and objectives of this series to submit research papers to Life Sciences, Society and Policy. As this is a thematic series (rather than a themed issue) there is no fixed deadline, and authors are explicitly encouraged to build on or critically respond to previous contributions. For further details or inquiries, please contact Hub Zwart, co-editor-in-Chief of LSSP (h.zwart@science.ru.nl).
